# A Novel Technique to Detect *EGFR* Mutations in Lung Cancer

**DOI:** 10.3390/ijms17050792

**Published:** 2016-05-23

**Authors:** Yuanbin Liu, Ting Lei, Zhiyu Liu, Yanbin Kuang, Jianxin Lyu, Qi Wang

**Affiliations:** 1 Department of Respiratory Medicine, the Second Hospital, Dalian Medical University, Dalian 116023, China; ybliu_301@163.com (Y.L.); kybkyb2@163.com (Y.K.); 2 Key Laboratory of Laboratory Medicine, Ministry of Education, Zhejiang Provincial Key Laboratory of Medical Genetics, Wenzhou Medical University, Wenzhou 325035, China; 3 Department of Thoracic Surgery, the Second Hospital of Dalian Medical University, Dalian 116023, China; leiting0612@sina.com; 4 Department of Urinary Surgery, the Second Hospital, Dalian Medical University, Dalian 116023, China; letter89@163.com

**Keywords:** RPA, ASA, SYBR, *EGFR* mutation, novel methodology, point-of-care test (POCT)

## Abstract

Epidermal growth factor receptor (*EGFR*) gene mutations occur in multiple human cancers; therefore, the detection of *EGFR* mutations could lead to early cancer diagnosis. This study describes a novel *EGFR* mutation detection technique. Compared to direct DNA sequencing detection methods, this method is based on allele-specific amplification (ASA), recombinase polymerase amplification (RPA), peptide nucleic acid (PNA), and SYBR Green I (SYBR), referred to as the AS-RPA-PNA-SYBR (ARPS) system. The principle of this technique is based on three continuous steps: ASA or ASA combined with PNA to prevent non-target sequence amplification (even single nucleotide polymorphisms, SNPs), the rapid amplification advantage of RPA, and appropriate SYBR Green I detection (the samples harboring *EGFR* mutations show a green signal). Using this method, the *EGFR* 19Del(2) mutation was detected in 5 min, while the *EGFR* L858R mutation was detected in 10 min. In this study, the detection of *EGFR* mutations in clinical samples using the ARPS system was compatible with that determined by polymerase chain reaction (PCR) and DNA sequencing methods. Thus, this newly developed methodology that uses the ARPS system with appropriate primer sets is a rapid, reliable, and practical way to assess *EGFR* mutations in clinical samples.

## 1. Introduction

Epidermal growth factor receptor (EGFR), a member of tyrosine kinase receptors, plays an important role in the regulation of cell proliferation, survival, and differentiation [1]. Upon ligand binding, as is the case with the epidermal growth factor (EGF), EGFR will form dimers to autophosphorylate the cytoplasmic tyrosine kinase domains and activate the EGFR signaling pathway [2]. Previous studies have shown that *EGFR* is overexpressed in a number of solid tumors, such as lung, breast, prostate, bladder, colon, head and neck, and ovarian carcinomas [3]. In non-small cell lung cancer (NSCLC), *EGFR* is overexpressed due to *EGFR* amplification or mutation [4,5,6]. In *EGFR* kinase domain mutations, 19Del(E746-A750) and L858R (the most prevalent mutations) account for nearly 90% of the *EGFR* mutations in NSCLC [7]. Patients with *EGFR*-mutated NSCLC have shown clinical responses to the orally administered EGFR inhibitor gefitinib, leading to a new era of the targeted therapy of human cancer [4,5,6]. Indeed, previous *in vitro* experiments have demonstrated that specific *EGFR* mutants have increased tyrosine kinase activity and possess a higher sensitivity to growth inhibition by tyrosine kinase inhibitors [8]. Thus, detection of *EGFR* mutations has become an important diagnostic procedure. To date, polymerase chain reaction (PCR) amplification of tumor specimens plus DNA sequencing has become a standard technique. Nevertheless, the molecular diagnosis methodology still greatly impedes this effect and remains a major obstacle for successful targeted therapy [9] because this technique requires sophisticated equipment and complex experimental procedures [10,11,12,13], including thermal cycling equipment and a DNA sequencer. Currently, an isothermal enzymatic DNA amplification system that includes nucleic acid sequence-based amplification [14], loop-mediated isothermal amplification (LAMP) [15], rolling circle amplification [16] and helicase-dependent amplification is commonly used [17]. Recently, the smart-amplification process method [18,19] has been developed to detect *EGFR* mutations, but the complex primer design, large equipment, and professional operators are disadvantages. In contrast, recombinase polymerase amplification (RPA) is a more advanced DNA-amplification method with a reaction temperature of 37 °C, easy primer design, and rapid amplification speed [20], yielding a large amount of product [21]. Moreover, the role of strand-exchange assisted by ATP hydrolysis is an interesting and useful method for gene amplification/detection. RPA can be applied to detect different DNA and RNA [22,23], but so far, it has not been used to detect human gene mutations.

Thus, in this study, we described a novel method based on allele-specific amplification (ASA), RPA, peptide nucleic acid (PNA), and SYBR Green I, which is called the AS-RPA-PNA-SYBR (ARPS) system, to identify *EGFR* mutations. This method could be used for the diagnosis of *EGFR* mutations and for the identification of patients suitable for targeted therapy. ASA/AS theory [24], RPA, PNA, and SYBR Green I were utilized in this system without any large equipment, sophisticated design of fluorescence-probe/primers, or lateral flow strips. The basis of this technology is to amplify mutated genomic DNA with the PNA technology specifically by inhibition of non-target sample amplification. After that, the recombinase polymerase-amplified products will generate fluorescence with SYBR Green I in order to visualize the mutant gene products. We anticipated that this method would be a reliable and cost-efficient method for the future screening of *EGFR* mutations that is consistent with Precision Medical development aspirations [25].

## 2. Results

### 2.1. Detection of EGFR Mutations in NSCLC Cell Lines and Comparison of Gold Standard PCR and Direct DNA Sequencing with Our Novel Detection Technique 

In this study, we detected *EGFR* mutations in cell lines using PCR and the direct DNA sequencing technique, and the data confirmed *EGFR* mutations in these NSCLC cell lines that matched with the ATCC cell line characteristics, *i.e.*, HCC-827 cells had an acquired *EGFR* mutation at the tyrosine kinase domain (E746-A750 deletion), while H-1975 cells harbored the heterozygous *EGFR* L858R mutation. In contrast, the A-549 cell line did not have any *EGFR* mutations.

**Figure 1:**
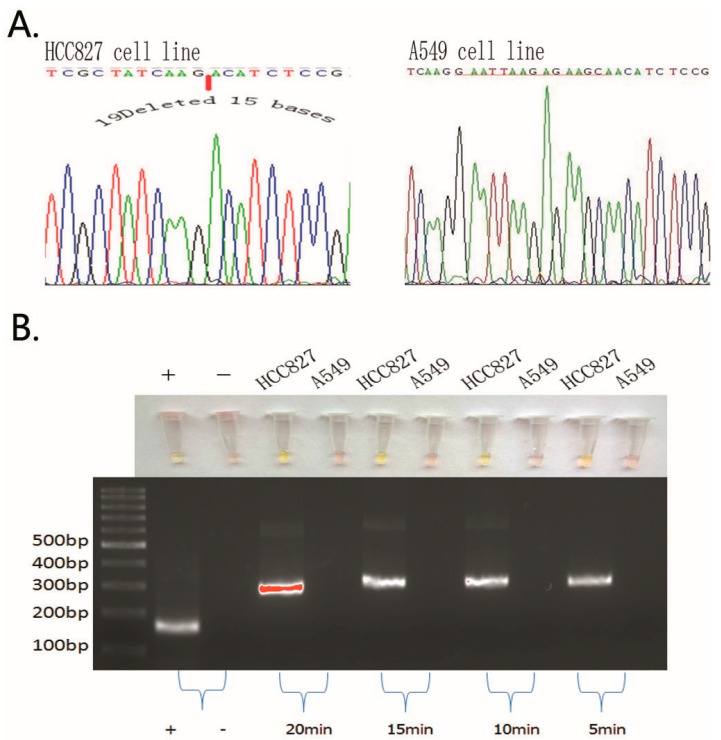
Comparison of the polymerase chain reaction (PCR) and DNA sequencing data with our visualization data using the ARPS method for detection of the epidermal growth factor receptor (*EGFR*)19Del(2) mutation in non-small cell lung cancer (NSCLC) cells. (**A**) The PCR and DNA sequencing data; (**B**) The specificity and timely sensitivity of the AS-RPA-PNA-SYBR (ARPS) method for detection of the *EGFR* 19Del(2) mutation. The “+” was the positive control in the recombinase polymerase amplification (RPA) reaction kit (143 bp in size), which was detected between 5 and 20 min. The mutated *EGFR* 19Del(2) band was 266 bp, and 300 ng of the DNA template was used. Results of ARPS were negative in red, positive in green.

**Figure 2:**
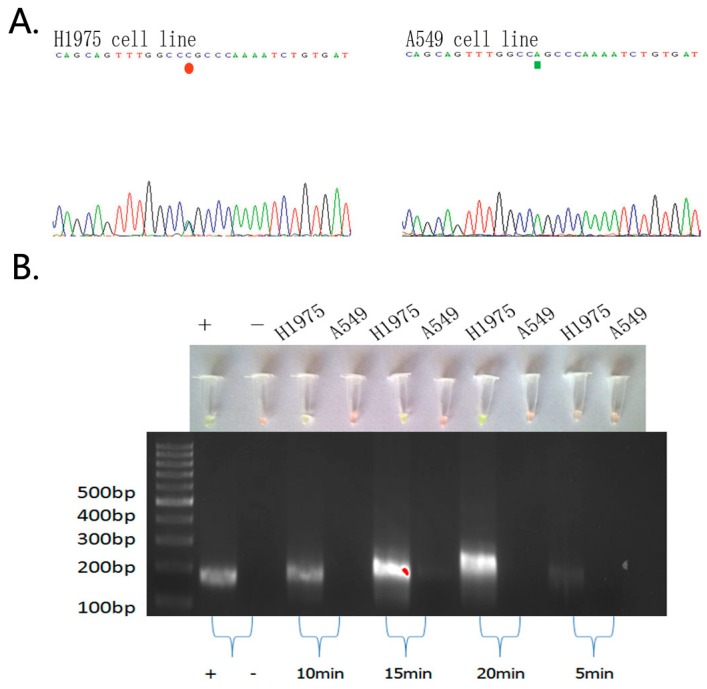
Comparison of the PCR and DNA sequencing data with our visualization data using the ARPS method to detect the *EGFR* L858R mutation in NSCLC cells. (**A**) The PCR and DNA sequencing data; (**B**) The specificity and timely sensitivity of the ARPS method for detection of the *EGFR* L858R mutation. The “+” was the positive control in the RPA reaction kit (143 bp in size), which was detected between 10 and 20 min. The target mutated band size was 201 bp, and 300 ng of the DNA template was used. Results of ARPS were negative in red, positive in green.

Next, we performed our novel technique to assess *EGFR* mutations in these cell lines and obtained similar results as the PCR and direct DNA sequencing data (Figure 1 and Figure 2).

### 2.2. Sensitivity of ARPS in Mixed-DNAs to Detect EGFR Mutations

**Figure 3:**
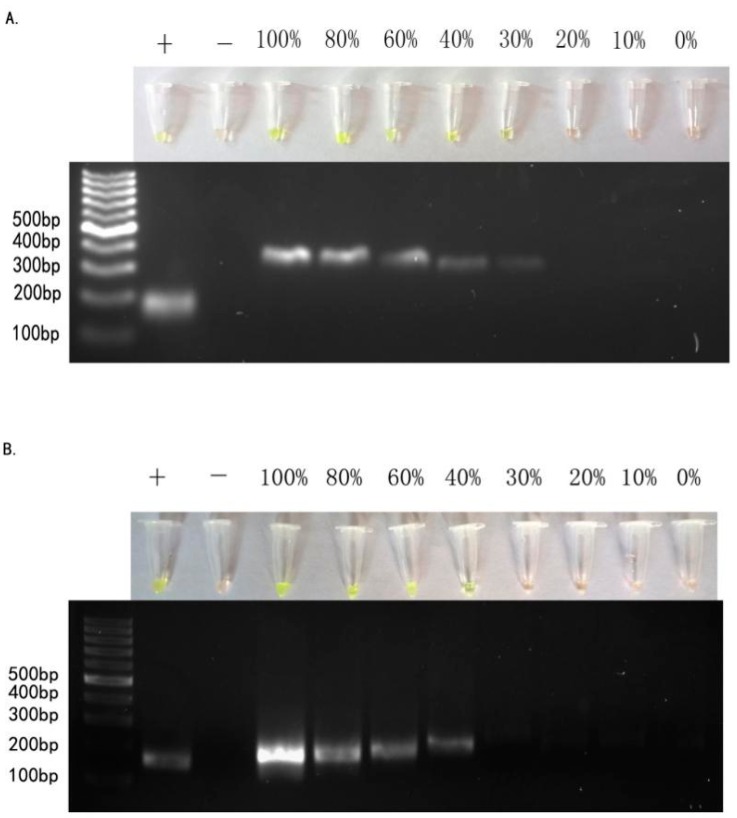
Sensitivity of the ARPS technique. (**A**) ARPS reaction using 19 Del(2) mutation-specific primers with serial dilutions of genomic DNA isolated from HCC-827 cells; (**B**) ARPS reaction using L858R point mutation-specific primers with serial dilutions of genomic DNA from H-1975 cells. Results of ARPS were negative in red, positive in green.

Tumor samples are frequently composed of numerous subpopulations of cancer cells. In addition to accurate puncture and sample extraction, detection of *EGFR* mutations must be capable of catching mutations in heterogeneous DNA samples. To test ARPS for this capability, we extracted genomic DNA from known *EGFR*-mutated tumor cell lines mixed with increasing amounts of wild-type DNA (the total amount of DNA was 300 ng) to assess the sensitivity of our method. In the serial dilution experiments with DNA samples from HCC-827, H-1975, and A549 cell lines, ARPS detected the *EGFR* 19Del(2) mutation and the *EGFR* L858R mutation with 30% and 40% of known *EGFR*-mutated genomic DNA samples, respectively, in 15 min (Figure 3).

### 2.3. Detection of EGFR Mutations in NSCLC Tissue Samples Using our Novel ARPS Method vs. the PCR and DNA Sequencing Method

**Figure 4:**
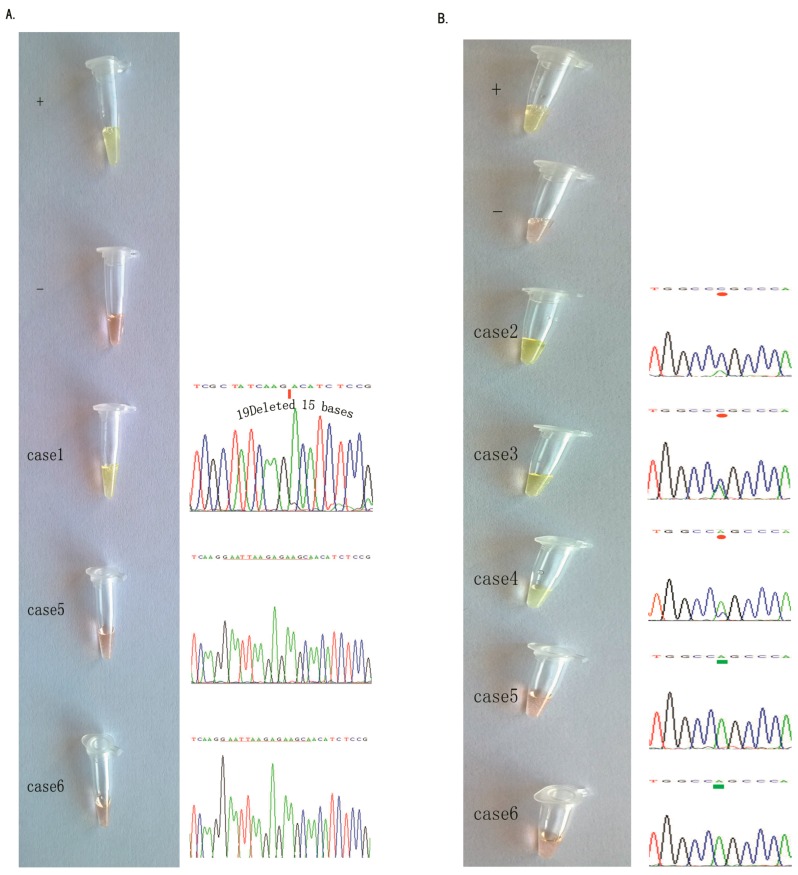
Comparison of the PCR and DNA sequencing data with our visualization data using the ARPS method to detect the *EGFR* mutation in NSCLC tissue specimens. (**A**) *EGFR* 19Del(2) mutation. The **left** panel shows our data, and the **right** panel shows the DNA sequencing data. From top to bottom, the quality control of the positive sample, the quality control of the negative sample (HCC-827 and A549 cell lines), one positive clinical tissue sample, and two negative clinical tissue samples are shown; (**B**) The *EGFR* L858R mutation. The **left** panel shows our data, and the **right** panel shows the DNA sequencing data. From top to bottom, the quality control of the positive sample, the quality control of the negative sample (H-1975 and A549 cell lines), three positive clinical tissue samples, and two negative clinical tissue samples are shown. Results of ARPS were negative in red, positive in green.

To further assess the *EGFR* mutations in the NSCLC tissue specimens, we collected 46 cases of NSCLC. Three cases were diagnosed with the *EGFR* L858R mutation and one with the *EGFR* 19Del(2) mutation using the amplification refractory mutation system (ARMS) in our hospital, and we reconfirmed these four mutated and two non-mutated samples in *EGFR* samples using the PCR and DNA sequencing method (Figure 4).

We then assessed the *EGFR* mutations in these six tissue samples using our novel method and found that after a 15-min amplification, we could mix SYBR in the cap and visualize the results (Figure 4), which were consistent with the PCR and DNA sequencing data.

### 2.4. Specific Considerations While Performing the ARPS Assay to Detect EGFR Mutations

**Figure 5:**
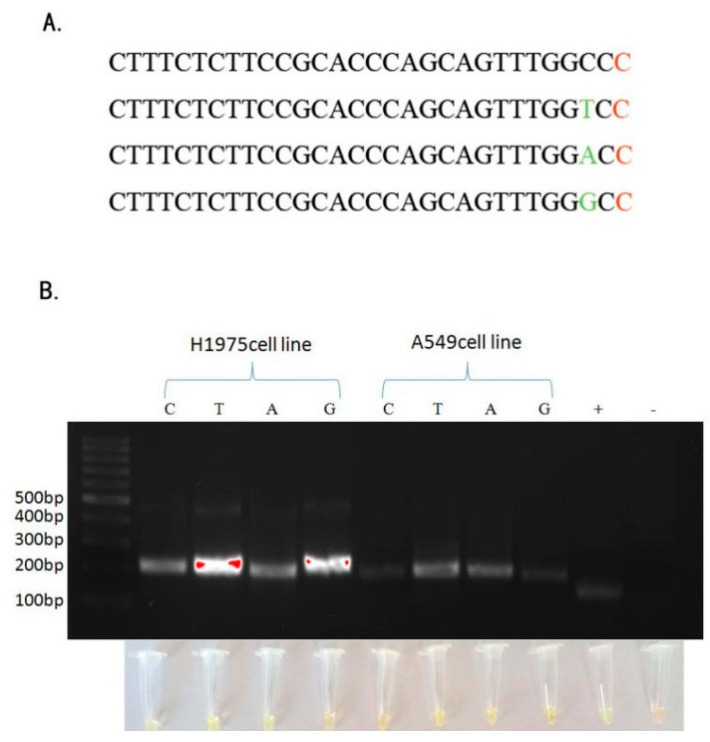
Primer sequences and agarose gel electrophoresis data. (**A**) The position of another mismatch base in the reverse primer (green letters) to detect *EGFR* L858R; (mutant base in red letter) (**B**) The agarose gel electrophoresis and ARPS results of the different mismatch bases. The band size was 201 bp, and the “+” indicates the positive control from the RPA reaction kit (143 bp). The amount of DNA used was 300 ng.

We first designed the specific ARPS primers to detect the *EGFR* 19Del(2) and L858R mutations (Table 1). However, in the selected set of *EGFR* L858R primers without the use of PNA, the agarose gel electrophoresis analysis showed very low specificity (Figure S1). The specificity was not improved even after the addition of the other base mismatched at the 3′-end of the reverse primers (Table 1 and Figure 5). The selected set of primers without mismatch fully corresponded to the mutated DNA sequences in the RPA experiment (Figure 1, Figure 2 and Figure 3). Moreover, the ethanol method received a satisfactory result with correct band positions, although our three trials of the column purification method failed.

Furthermore, SYBR was really sensitive at detecting double-stranded DNA, and the minimum detection limit was 20 pg of double-stranded DNA under 254-nm ultraviolet (UV) light. However, the use of UV light should be avoided with this method because both positive and negative results will show fluorescent green. Moreover, the amount of DNA sample should be less than 2 μg, and 2 μL of 10 mM each primer should be used; otherwise, the experimental results cannot be visualized by the naked eye. Thus, in our optimized SYBR detection system (Figure S2), the template of genomic DNA was 300 ng with 2 µL of each primer (10 mM) and 1 μL of 200× SYBR per 20 μL of RPA product.

As for the specificity and timely sensitivity of the ARPS method at detecting the *EGFR* 19Del(2) mutation (deletion of 15 base pairs), the identification of specific DNA fragments was achieved by this method from 5 to 20 min, and the ARPS results were consistent with the sequencing results (Figure 1A). After the RPA reactions were complete, 30 μL of the product was transferred into a new 1.5-mL Eppendorf tube, and 1 μL of 200× SYBR was added to the former tube within 1 min. In addition, we purified the 30 μL of product using our ethanol method and performed agarose gel electrophoresis to confirm the band position (Figure 1B). When the ARPS reaction reached more than 40 min, a false-positive band appeared.

To detect the *EGFR* L858R mutation, we used the selected set of primers and obtained specific DNA fragments between 10 and 20 min, as shown by electrophoresis (Figure 2).

## 3. Discussion

In the current study, we developed the ARPS method to detect *EGFR* mutations with two sets of specifically designed primers capable of recognizing a 15-bp deletion of *EGFR* and an *EGFR* SNP-type mutation in both NSCLC cell lines and tissue samples. We were able to successfully assess the *EGFR* 19Del(2) mutation in 5 min and the *EGFR* L858R mutation in 10 min in NSCLC cell lines with the naked eye. Moreover, we also successfully detected these mutations in NSCLC tissue samples. Compared to the PCR and DNA sequencing method, our newly developed ARPS method apparently has several advantages, including a shorter analysis time, low cost, and more convenient method, over the conventional DNA sequencing method. In addition, ARPS allows us to detect *EGFR* mutations in 30% to 40% of targeted mutant alleles in genomic DNA samples without any background amplification, which is similar to or better than DNA sequencing.

While performing this assay, we utilized the SYBR-assisted detection method and determined the appropriate SYBR concentration to be 1 μL of 200× SYBR per 20 μL of RPA product. Meanwhile, we set the appropriate amount of DNA template to 300 ng because too much DNA template would jeopardize the SYBR detection and too little would also affect sensitivity and produce primer-dimers. After the RPA reaction, mixing SYBR in the cap resulted in the feasibility of SYBR Green I detection [26]. Furthermore, the ethanol method was more efficient and convenient to purify the RPA products for electrophoresis. In contrast, using the column method, DNA could not be isolated according to the manufacturer’s protocol of the EasyPure^®^ PCR Purification Kit (TransGen Biotech, Beijing, China). We speculate that this may be because the DNA-protein complex in the reaction could not adsorb to the adsorption column or because the complex could not elute from the column. So, either collection of the eluted liquid from the bottom tube (as suggested by the manufacturer) or the addition of preheated solution EB (triple-distilled water) into the column for 1 min was not able to recover DNA for agarose gel electrophoresis.

Furthermore, to detect the 15-bp deletion of *EGFR*, we utilized both SYBR detection and electrophoresis to obtain visible bands, with an absolute accuracy between 5 and 20 min. The RPA reaction played a key role to shorten the detection time. However, when the RPA reaction was finished, the addition of SYBR to the RPA reaction mixture needed to be rapid because the RPA reaction is continuous at room temperature; thus, a reaction time of 40 min or more should be avoided. Furthermore, to detect the single base mutation of *EGFR*, our newly developed method used both SYBR detection and electrophoresis, and excellent results were obtained within 10 and 20 min.

In addition, other research groups have explored the MutS [18,19,27] protein or PNA probe [28] to reduce the background DNA amplification in LAMP or the PCR method. In the current study, we tried the MutS protein (Gene check Inc., Fort Collins, CO, USA) to prevent background amplification of the single base mutation in the RPA reaction, but the data were ambiguous and the reason is unclear. Furthermore, the use of the RPA reaction to detect the *EGFR* mutation has been reported by end-point fluorescence detection, lateral flow strips, or microfluidic chips [29,30,31], but there is no report thus far showing this ARPS system. 

However, our currently described novel method does have some limitations; for example, for each *EGFR* mutation, we need to optimize the methodology to obtain specificity and sensitivity, while each *EGFR* mutation also needs a specific primer set. Furthermore, our current study only assessed a limited number of clinical tissue samples, and more cases should be assessed in a future study for association with the clinical outcome of EGFR tyrosine kinase therapy. Although the sensitivity of ARPS is relatively lower than other methods, the setup of ARPS is very simple and easy. We could detect *EGFR* mutations within 15 min, and the specificity was robust without non-targeted sequence amplification. The unique primer design, background suppression technology, and 37 °C isothermal nature of this assay could provide a practical way for bedside usage or POCT in molecular diagnosis. Our newly developed ARPS method is useful to detect *EGFR* mutations, especially in un- or underdeveloped regions and countries because such areas may not have instrumentation for DNA sequencing and purification procedures are not required to detect the products. 

## 4. Materials and Methods

### 4.1. Cell Lines and Culture

The human non-small cell lung cancer cell lines NCI-H1975, HCC-827, and A-549 were obtained from the American Type Culture Collection (Manassas, VA, USA) and maintained in RPMI-1640 medium (Gibco, Gaithersburg, MD, USA) supplemented with 10% fetal bovine serum (HyClone, Logan, UT, USA), 100 U/mL penicillin, and 100 U/mL streptomycin at 37 °C in a humidified incubator containing 5% CO_2_ and 95% air. NCI-H1975 cells harbor the *EGFR* exon 21 L858R point mutation, HCC-827 cells harbor the *EGFR* exon19 deletion(2), and A-549 cells contain wild-type *EGFR* with these two-targeted DNA sequences.

### 4.2. NSCLC Tissue Samples

We collected surgical tissue samples from 46 consecutive patients who were treated at the Second Hospital of Dalian Medical University (Dalian, China) in 2015. All patients were diagnosed with NSCLC according to the *International Association for the Study of Lung Cancer* lung cancer staging protocol [32]. This study was approved by the Second Hospital of Dalian Medical University, and a written informed consent form was obtained from each patient before participating in this study. Of these 46 patients, 10 cases were subsequently treated with gefitinib. This cohort of patients included 25 females and 21 males with an age at diagnosis ranging from 42 to 87 years old (median of 66 years old). After surgical resection, all tumor samples were immediately snap-frozen and stored at −80 °C until use.

### 4.3. Genomic DNA Extraction and Detection of EGFR Mutations Using PCR and DNA Sequencing

Genomic DNA was extracted from NCI-H1975, HCC-827, and A-549 cells as well as patient tissues using DNAiso Reagent (Takara, Dalian, China), according to the manufacturer’s instructions. The DNA concentrations were measured using the 260/280 ratio obtained with a spectrophotometer and adjusted to 150 ng/μL. The samples were stored at −20 °C until use.

Detection of *EGFR* mutations in these three NSCLC cell lines and tissue samples was performed by PCR amplification with a PCR amplification kit (TransGen, Beijing, China), followed by DNA sequencing. The primers for amplification of *EGFR* 19Del(2) and L858R regions were the same as described in our previous study (Table 2). The PCR mixtures contained 2 μL of genomic DNA, 1 μL of each primer (100 μM), 25 μL of 2× EasyTaq^®^PCR Supermix, and 21 μL of ddH_2_O; the thermal cycling conditions were set to an initial denaturation step at 94 °C for 5 min and then 30 cycles of 94 °C for 30 s, 55 °C for 30 s, and 72° C for 20 s, and a final extension at 72 °C for 5 min. The PCR products were then sequenced by using an ABI PRISM 3730 Genetic Analyzer^®^ (ABI, Austin, TX, USA), and the data were analyzed by using ABI PRISM SeqScape Software Version 5.2.0^®^ (ABI, Austin, TX, USA). The experiments were repeated three times.

### 4.4. The Novel Technique to Detect EGFR Mutations

#### Principle of RPA primer design

RPA primers were designed according to the instrument guide and principles, *i.e.*, primer length of 30–35 bases and preferred amplicons of 80–400 bp long. In addition, the 3′-primer end for both the forward and the reverse primers should avoid the third codon genome, although it would be fine for 5′-primers.

To distinguish the mutated sequence from the wild-type one, we designed primers with the help of ASA/AS theory. For the *EGFR* 19Del(2) mutation, we removed the deleted 15 bp from the forward primer and tried to make the missing portion near the primer 3′-end. For the *EGFR* L858R mutation, we put the single mutated base at the 3′-end of the reverse primer and added an additional mismatch (another three dNTPs) two bases from its 3′-end. The specificity of these primer sequences was confirmed by alignment to the human genome database using BLAST.

### 4.5. Probe Design with PNA to Prevent Non-Target Sequence Amplification 

We designed a probe using the peptide nucleic acid from Beijing Search Co., Ltd. (Beijing, China), which is a type of polypeptide skeleton instead of the main chain of the sugar phosphate DNA analogues. Compared to the same sequence of DNA primers, PNA-DNAs have a stronger binding force and a higher melting temperature, and PNA cannot be detected by SYBR Green I, even under UV light. Thus, we added PNA to combine with the non-target sequence as a probe, ensuring that the RPA could only amplify the mutated sequence. The melting temperature value of the designed PNA probe was found at the web site [33].

### 4.6. Biosensor SYBR Green I 

SYBR Green I (Beijing Solarbio Science and Technology Co., Ltd., Beijing, China) is a cost-effective molecular probe to detect double-stranded or single-stranded DNA during PCR amplification. When the level of the DNA products passes the threshold level, the prescribed concentration of SYBR would turn from the original orange color to green as visualized by the naked eye.

### 4.7. ARPS Reactions

For *EGFR* 19Del(2), a total volume of 50 μL of RPA mixture containing 12 μL of tri-distilled water, 2 μL of each forward and reverse primer, 2 μL (300 ng, corresponding to the SYBR detection mechanism) of genomic DNA, and 29.5 μL of rehydration buffer was mixed and added with a freeze-dried reagent pellet from the TwistAmp^®^ (TwistDX, Cambridge, UK) basic kit. The mixture was thoroughly vortexed and then added with 2.5 μL of 280 mM magnesium acetate solution.

For EGFR L858R, the only difference in SNP amplification was the PNA combining process, *i.e.*, the ARPS reaction contained 10 μL of tri-distilled water, 2 μL of PNA, and 2 μL of genomic DNA in the Eppendorf tube (the PNA concentration was twice that of the primer concentration). The combining process was set to 99 °C for 2 min and 66 °C for 2 min. The primers, the rehydration buffer, amplification enzymes, and magnesium acetate solution were then added to the reaction, accordingly, the same as those of EGFR 19Del(2).

### 4.8. Agarose Gel Electrophoresis of RPA Products 

After the RPA reaction was completed, we purified the products using two methods, *i.e.*, (i) A purification column (EasyPure^®^ PCR purification Kit) and (ii) Use of ethanol to remove the RPA reaction buffer. For the purification column method, the DNA extraction procedure was performed according to the manufacturer’s protocol (TransGen Biotech, Beijing, China). For the ethanol method, 30 μL of the RPA product was added to 100 μL of 99.8% ethanol and centrifuged at 12,000 rpm for 5 min. Next, the supernatant was discarded, and this procedure was repeated twice to purify the RPA products. After that, 30 μL of tri-distilled water was added to the tube for agarose gel electrophoresis in a 1.5% gel.

### 4.9. Assessment of Specificity and Sensitivity of the ARPS Assay for EGFR Mutations

The specificity of the RPA detection assay was assessed by using the DNA extracted from the non-*EGFR* mutated A549 cell line and the target-mutated HCC-827 and H1975 cell lines, with the *EGFR* 19Del(2) and *EGFR* L858R mutations, respectively. Specifically, 300 ng of genomic DNA was used to be consistent with SYBR detection color rendering. After the RPA reactions were complete, 30 μL of the product was added into a new 1.5-mL Eppendorf tube, and then 1 μL of 200× SYBR was added to the remaining 20 μL of product within 1 min to prevent the RPA reaction from continuing. After the other 30 μL of product was purified using the ethanol method, agarose gel electrophoresis was conducted to confirm the band position.

### 4.10. Quality Control

We utilized the control from the TwistAmp^®^ basic kit (TwistDx Inc., Cambridge, UK) for the cell line quality control, and the expected product size was 143 bp (TwistAmp^®^). For the tissue samples, genomic DNA from cell lines with known positive and negative *EGFR* mutations was used as controls.

## Figures and Tables

**Table 1:** RPA primers used to detect *EGFR* 19Del(2) and L858R mutations.

*EGFR* Mutation Type Primers Amplicon (bp)
19Del(2)FP 5’-GTGAGAAAGTTAAAATTCCCGTCGCTATCAAGACATCTC-3’ 266
19Del(2)RP 5’-GATACCAGCATGGGAGAGGCCAGTGCTGTCTCTAAG-3’
L858R FP 5’-CTGAATTCGGATGCAGAGCTTCTTCCCATGA-3’ 201
L858R RP 5’-CTTTCTCTTCCGCACCCAGCAGTTTGGCCC-3’
PNA 5’-GCCAGCCCAA-3’

FP, Forward Primer; RP, Reverse Primer; PNA, peptide nucleic acid.

**Table 2:** PCR primers used to detect *EGFR* mutations

EGFR Mutation Type Primers Amplicon (bp)
19Del FP 5’-AACGTCTTCCTTCTCTCTCTGTCA-3’ 135(Mut)/150(Wild)
19Del RP 5’-CCACACAGCAAAGCAGAAACT-3’
L858R FP 5’-CTGAATTCGGATGCAGAGCTT-3’ 291
L858R RP 5’-CTAGTGGGAAGGCAGCCTGGT-3’

FP: Forward Primer, RP: Reverse Primer, Mut: mutation type, Wild: wild type.

## Supplementary Materials

Supplementary materials can be found at http://www.mdpi.com/1422-0067/17/5/792/s1.
