# Supplementary material for: A Novel Technique to Detect EGFR Mutations in Lung Cancer
Source: Int J Mol Sci. 2016 May 23;17(5):792. doi: 10.3390/ijms17050792 (PMC4881608; doi:10.3390/ijms17050792)
Supplement: Supplementary file 1 [file ijms-17-00792-s001.pdf]

Supplementary Materials: A Novel Technique to Detect *EGFR* Mutations in Lung Cancer

Yuanbin Liu, Ting Lei, Zhiyu Liu, Yanbin Kuang, Jianxin Lyu and Qi Wang

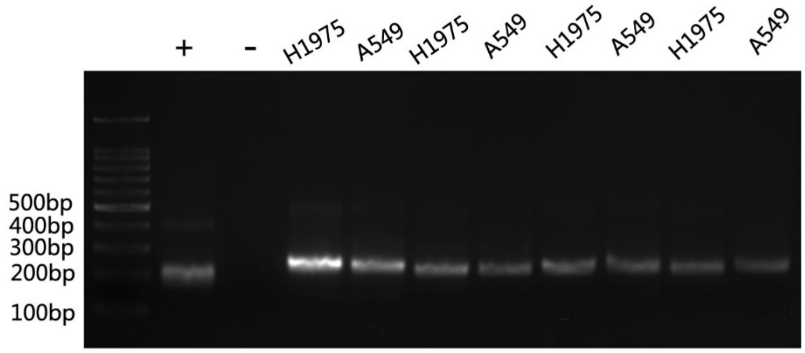

Figure S1. Primer set without PNA for detecting the L858R mutation in 10 min.

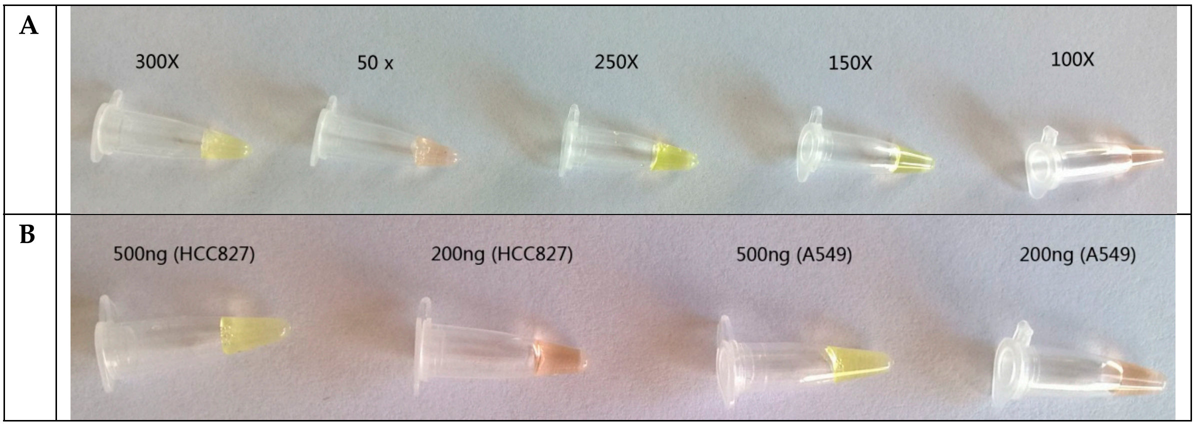

Figure S2. (**A**) Optimization of the SYBR detection system. The products of the ARPS system for detecting the *EGFR*L858R mutation in the HCC827 cell line (300ng DNA), which harbors the 19Del mutation, in 15 min. From left to right, the concentration of SYBR was 1 μL of 300×, 50×, 250×, 150×, and 100× per 20 μL of product. We chose 1 μL of 200× SYBR per 20 μL of RPA product for the experiment; (**B**) Optimization of the SYBR detection system. The concentration of SYBR was 1 μL of 200× SYBR per 20 μL of RPA product. The products of the ARPS system for detecting the *EGFR*L858R mutation in the HCC827 cell line (500 ng/200 ng DNA) and the A549 cell line (500 ng/200 ng DNA) in 15 min. We chose 300 ng of template as in the experiment shown in Figure S1.
